# Survey of UK radiology trainees in the aftermath of ‘Modernising Medical Careers’

**DOI:** 10.1186/1472-6920-12-93

**Published:** 2012-10-02

**Authors:** Grant Mair, Fiona Ewing, John T Murchison

**Affiliations:** 1 Department of Radiology, Royal Infirmary of Edinburgh, 51 Little France Crescent, Edinburgh, EH16 4SA, UK; 2 Department of Radiology, Western General Hospital, Edinburgh, UK

**Keywords:** Radiology, Training, Modernising, Medical, Careers, MMC, Undergraduate, Postgraduate, Survey

## Abstract

**Background:**

Following implementation of Modernising Medical Careers (MMC) in the UK, potential radiology trainees must decide on their career and apply sooner than ever before. We aimed to determine whether current trainees were sufficiently informed to make an earlier career decision by comparing the early radiology experiences of Traditional and Foundation Trainees.

**Methods:**

344 radiology trainees were appointed through MMC in 2007/08. This cohort was surveyed online.

**Results:**

Response rate was 174/344 (51%). Traditional Trainees made their career decision 2.6 years after graduation compared with 1.2 years for Foundation Trainees (57/167, 34%). Nearly half of responders (79/169, 47%) experienced no formal radiology teaching as undergraduates. Most trainees regularly attended radiology meetings, spent time in a radiology department and/or performed radiology research. Many trainees received no career advice specific to radiology (69/163, 42%) at any point prior to entering the specialty; this includes both formal and informal advice. Junior doctor experiences were more frequently cited as influencing career choice (98/164, 60%). An earlier career decision was associated with; undergraduate radiology projects (-0.72 years, p = 0.018), career advice (-0.63 years, p = 0.009) and regular attendance at radiology meetings (-0.65 years, p = 0.014).

**Conclusion:**

Early experience of radiology enables trainees to make an earlier career decision, however current radiology trainees were not always afforded relevant experiences prior to entering training. Radiologists need to be more proactive in encouraging the next generation of trainees.

## Background

Radiology training in the UK has traditionally been undertaken following an initial period of postgraduate work. Candidates for radiology generally already had several years of clinical experience in other specialties and because entry was highly competitive, most had voluntarily opted to pursue an alternative postgraduate qualification. Turner et. al.’s Medical Careers Research Group has over a 30 year period followed-up graduates from all UK medical schools. They found that nearly two thirds of currently practicing radiologists made their career decision three years after graduation, while only 20% had decided in the first postgraduate year [1].

Modernising Medical Careers (MMC) was a government led overhaul of all postgraduate medical training in the UK instigated in 2005 with the introduction of Foundation Training programs. In 2007, entry into higher specialist training was also revamped. MMC was an attempt to streamline the process of moving doctors from basic to specialty training [2]. Prior to MMC, medical graduates in the UK would undertake one generic year of pre-registration training (as Pre-Registration or Junior House Officers) followed by a variable number of years (but usually at least two) in basic training posts (Senior House Officers). Speciality training would therefore usually begin after a minimum of three years from graduation. Under MMC, Foundation Training totals two years and following a single phase of nationally coordinated interviews, Specialty Training (ST) begins immediately thereafter for some disciplines (including radiology). Application for an ST radiology training program is usually submitted in December of the second year of Foundation Training and therefore needs to be completed approximately 16 months after graduation; this is earlier than ever before. Accordingly, contemplating a career in radiology and building a portfolio of relevant experience must begin as an undergraduate, a process that requires adequate radiology exposure not only clinically, but also educationally and in a research context. It is our supposition that current undergraduate curricula and early postgraduate training in the UK do not adequately prepare trainees to make an informed decision for entering a career in radiology through MMC.

The aim of this survey was to determine whether potential trainees to radiology are sufficiently experienced to make an informed career decision following shortening of the generic pre-specialty training time as imposed by MMC and if necessary, identify methods to improve early access to radiology for potential trainees. By assessing trainees’ early experiences of radiology and comparing responses obtained from *Foundation Trainees* (those who completed a Foundation Program as implemented by MMC) with responses returned by *Traditional Trainees* (those who did not complete a Foundation Program and are representative of pre-radiology training prior to MMC) we hoped to ascertain how this major change in postgraduate training is affecting those on whom it has been imposed.

## Results

### Demographics

**Figure 1:**
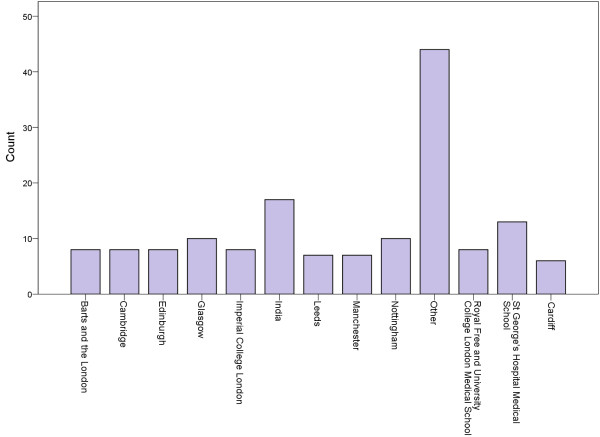
**Medical schools attended by those surveyed.** Other (n) = Aberdeen (3), Birmingham (1), Bristol (4), Egypt (1), Estonia (1), Germany (1), Greece (1), Guy’s London (4), Iran (3), Iraq (1), Jordan (1), Leicester (2), Liverpool (2), Nepal (1), Newcastle (1), Oxford (4), Pakistan (2), Queen’s Belfast (3), Saint Andrews (1), Sheffield (3), South Africa (2), Southampton (1), West Indies (1).

174 responses were obtained giving a response rate of 51%. From these, 154/174 (89%) surveys were completed in their entirety. Respondents comprised 60/157 (38%) first and 97/157 (62%) second year trainees; 96/158 (61%) were male. Most trainees were less than 30 years of age at the time of survey (108/158, 68%). A significant proportion completed a Foundation Program (57/167, 34%). All 16 UK training schemes (deaneries) are represented; the mean response rate was 45% per deanery. Trainees came from a range of 38 different medical schools. The most frequently encountered medical schools are displayed in Figure 1. International medical schools, i.e. those outside of the UK (32/154, 21% of total) were grouped by country; 12 other countries are represented, with the largest number from India (17/154, 11% of total trainees surveyed).

International medical graduates showed no significant difference in the timing of their decision to train in radiology. A higher percentage of these trainees have a postgraduate qualification, but this difference was not significant. International graduates were less likely to have completed a Foundation Program (8/32, 25% versus 45/122, 37% for those trained in the UK). International graduates were significantly more likely to be older (p < 0.001). No other differences were identified between radiology trainees who had UK based compared with overseas undergraduate medical training. The survey made no assessment of the location of any postgraduate, pre-radiology training.

### Choosing radiology

Foundation Trainees made their decision to enter radiology training 1.2 years after graduation; these trainees were obliged to apply during their second postgraduate year. This compared with 2.6 years for Traditional Trainees who were not obliged to apply at any particular time in their training. Twice as many Foundation Trainees made their decision at the undergraduate level (Table 1).

**Table 1:** Timing of career decision

**Year of decision**	**Foundation trainee**	**Traditional trainee**
	**(n = 57)**	**(n = 110)**
	**Percentage of trainees (%)**	
Undergraduate	25	13
Postgraduate Year 1	36	8
Postgraduate Year 2	39	29
Postgraduate Year 3	-	21
Postgraduate Year 4	-	16
Postgraduate Year 5 or Later	-	13
Years from Graduation (mean)	1.2 years	2.6 years

Comparing those who completed a Foundation Program (Foundation Trainee) with those trainees who did not (Traditional Trainee).

Prior to applying for radiology, 115/174 (66%) trainees had alternative career plans and 78/174 (45%) concurrently applied to another specialty. Foundation Trainees were significantly less likely to have alternative career plans (χ^2^ = 5.34, p = 0.021, df = 1, OR = 0.41 [95% CI 0.19-0.90]). Completion of the Foundation Program however, was not associated with whether trainees applied to specialties other than radiology during the MMC process. Foundation Trainees were offered significantly more training posts through MMC (p = 0.014). The majority of trainees (141/173, 82%) obtained postgraduate qualifications prior to entering radiology. Traditional Trainees were more likely to have a postgraduate qualification (χ^2^ = 39.9, p < 0.001, df = 1, OR = 0.05 [95% CI 0.01-0.22]), and for this qualification to be completed (χ^2^ = 21.19, p < 0.001, df = 1, OR = 0.14 [95% CI 0.06-0.36]). Surgery was the most common alternative career path and the majority of postgraduate exams completed across the cohort were surgical (90/141, 64%). Medical exams prior to radiology were less common (39/141, 28%).

### Early experience of the specialty

Table 2 shows the uptake of relevant clinical experiences by participants prior to entering radiology training.

**Table 2:** Early clinical experiences of radiology cited by trainees

**Experience**	**Uptake n (%)**
Regular Attendance at Radiology Meetings (at least weekly)	145/167 (87)
Radiology Research/Audit	131/163 (80)
Formal Time in Radiology Department (e.g. Taster Week)	107/167 (64)
Career Advice Specific to Radiology	94/163 (58)
Undergraduate Radiology Tuition	90/169 (53)
Undergraduate SSM/Student Elective Related to Radiology	38/88 (43)
Intercalated BSc Related to Radiology	21/79 (27)
Radiology Based Rotation within Foundation Program	6/57 (11)

*SSM* - Special Study Module.

Nearly half of the trainees surveyed (79/169, 47%) received no undergraduate radiology teaching (defined as ‘session taken by a radiologist where the primary focus was image interpretation’), with only 42/169 (25%) having more than 10 sessions in total. Those with no undergraduate tuition were significantly more likely to have graduated earlier (p = 0.025). Undergraduate projects or student electives commonly involved at least some radiology (either primarily based in radiology, or projects based in anatomy, pathology or medical physics with radiological input); an intercalated BSc associated with radiology was less common.

As junior doctors, most radiology trainees (145/167, 87%) attended local radiology meetings weekly, with 10/167 (6%) attending less frequently than once a month or never. Only 6/57 (11%) of Foundation Trainees had a radiology based rotation as part of their Program. Nearly two thirds of trainees spent formal time in a radiology department prior to applying.

Most trainees performed radiology based research or audit prior to applying (131/163, 80%); audit was most common (103/163, 63%). Many trainees received no career advice specific to radiology (69/163, 42%); for those who did, informal discussions were most common (69/163, 42%) while formal advice and departmental open days were rare (13/163, 8% and 12/163, 7%, respectively).

Foundation Trainees were more likely to receive undergraduate tuition in radiology (χ^2^ = 18.77, p < 0.001, df = 1, OR = 0.41 [95% CI 0.21-0.82]) and to undertake a taster week (χ^2^ = 7.12, p = 0.008, df = 1, OR = 0.35 [95% CI 0.15-0.82]). There was no significant difference in attendance at departmental meetings, on uptake of research opportunities or career advice for Foundation compared with Traditional Trainees.

For those who cited an undergraduate experience as influencing eventual career choice (59/166, 36%), an SSM (Special Study Module) or elective was most common. Experiences as a junior doctor were more frequently cited as influencing career choice (98/164, 60%), especially departmental meetings/day to day work (37/164, 23%) and Taster Sessions (31/164, 19%). Those who obtained career advice (‘did you attended a career open day for radiology, or obtain career advice specific to radiology (including informal discussions)?’) cited this as more influential in their decision than research experience; 27/129 (21%) of those who obtained career advice found that this influenced their career decision. The influence of both undergraduate and postgraduate experiences was the same for both Traditional and Foundation Trainees.

### Factors affecting timing of career decision

The impact of various radiology experiences on timing of career decision was assessed; see Table 3. To limit confounding, results are compared between those who did and those who did not complete the Foundation Program. All of the experiences highlighted in Table 2 were found to affect the timing of a career decision; many of these associations were statistically significant.

**Table 3:** Association between uptake of various clinical experiences and timing of career decision

**Experience**	**Effect on decision in years**	
	**Foundation trainee**	**Traditional trainee**
Radiology Audit or Research (Including Intercalated BSc)	-0.29	-0.24
Regular Attendance at Radiology Meetings	-0.65 (0.014)	-0.51
	95%CI -0.15 to -1.16	
Formal Time in Radiology Department (e.g. Taster Week)	No difference	-0.48
Career Advice Specific to Radiology	-0.63 (0.009)	-0.38
	95%CI -0.18 to -1.10	
Undergraduate Radiology Tuition	No difference	-0.26
SSM/Elective Related to Radiology	-0.72 (0.018)	-0.20
	95%CI -0.14 to -1.30	
Alternative Career Plan	0.32	1.47 (<0.001)
		95%CI 0.83 to 2.11

Results provided for both those who did (Foundation Trainee) and those who did not (Traditional Trainee) complete a Foundation Program. A negative number indicates an earlier decision. If statistically significant, p-values are provided in parentheses. SSM- Special Study Module.

As expected, an alternative career plan delayed the decision to train in radiology; this was most marked and statistically significant for Traditional Trainees, delaying the decision by 1.47 years.

Foundation Trainees who regularly attended radiology meetings (most days or most weeks), obtained career advice specific to radiology or undertook an SSM/student elective related to radiology were more likely to decide to train in radiology sooner. Similar trends were identified for Traditional Trainees, although these trends were not statistically significant. Performing radiology based research or audit (including an intercalated BSc), spending formal time in a radiology department, or obtaining undergraduate tuition in radiology - although influential in the career decision making process - did not lead to a statistically significant effect on decision timing, although there were also trends to an earlier decision here.

### Outcome of decision to train in radiology

Trainees who did not feel adequately prepared to make the decision to enter radiology training had less time between graduation and starting training than those who were adequately prepared but this difference was not significant. There was no association between the Foundation Program and whether or not trainees felt ready to begin radiology. Similarly, the career stage at which the decision was made was not associated.

**Figure 2:**
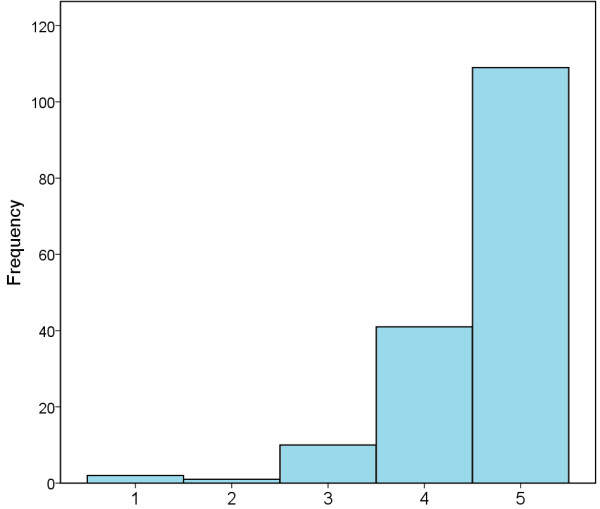
**Job satisfaction in radiology for those surveyed.** 1 = Planning to Change Career, 2 = Unhappy, 3 = Not Sure, 4 = Happy, 5 = Delighted.

**Figure 3:**
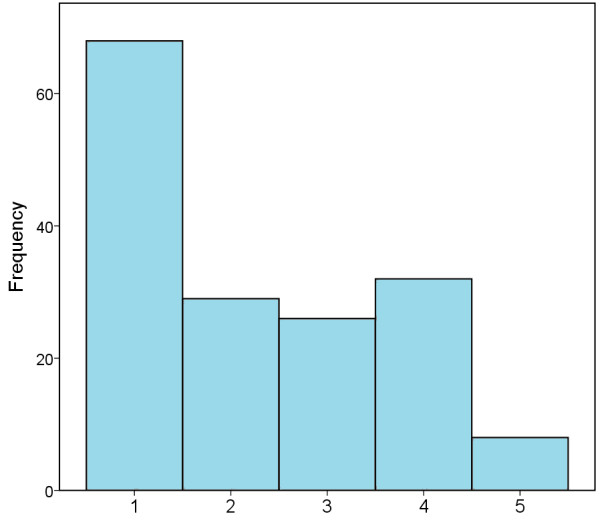
**Satisfaction scores for the MMC process in those surveyed.** 1 = Very Disappointed, 2 = Mildly Disgruntled, 3 = Indifferent, 4 = Happy, 5 = Delighted.

The vast majority of respondents (147/162, 91%) felt adequately prepared and ready to embark on their radiology career at the point of entry. Job satisfaction was high in the cohort; ranked on a scale of 1 (unhappy) to 5 (delighted), the mean result was 4.6 (Figure 2). The vast majority of trainees (128/163, 79%) obtained a training post in their first (101/163, 62%) or second (27/163, 17%) choice deanery through MMC. There was however, dissatisfaction with the process; on a scale of 1 (very disappointed) to 5 (delighted) the mean was 2.3 (Figure 3).

Overall satisfaction with radiology was not related to whether or not trainees completed the Foundation Program, or whether they frequently attended departmental meetings or undertook a taster week. There was a trend toward higher satisfaction scores in radiology for those who had career advice though this was not significant. Satisfaction with MMC was not related to decision timing, neither was satisfaction with radiology as a career. Those trainees who applied to other specialties were more satisfied with the MMC process overall (p = 0.006) but this did not affect satisfaction with radiology as a career. Foundation Trainees were more satisfied with the MMC process (p < 0.001).

## Discussion

With this survey, we have demonstrated that following the changes to specialty training imposed by MMC (Modernising Medical Careers) in 2007 applicants to UK radiology training must make a career decision much sooner than ever before. Trainees choose radiology based on undergraduate and postgraduate experiences of the specialty and these experiences are associated with an earlier career decision. Unfortunately, trainees in our survey had limited experience of the specialty at an undergraduate level and insufficient formal insight of the job in their early postgraduate years. The situation seems to be improving but radiologists and medical schools need to be more proactive in exposing the next generation of trainees to the specialty.

### Timing of a career decision and the impact of MMC

Following implementation of MMC changes to specialist training, radiology applications must now be submitted within the second postgraduate year. Prior to this, most trainees needed 3 years just to decide on a career in radiology [1]. It should be noted that the basic entry requirement imposed by the Royal College of Radiologists (RCR) has not changed during the MMC transition period; trainees are still required to have obtained a minimum two years of ‘appropriate clinical experience’ [3,4] i.e. MMC affords potential radiology trainees only that minimum clinical experience. In our survey, *traditionally* trained applicants to radiology (completed undergraduate medical training before 2005) made their career decision 2.6 years after graduation. As expected, Foundation Trainees (graduated 2005 or later; these trainees represent the new style of basic medical training in the UK) decided nearly 18 months sooner. A significant number of these trainees decided in the same year they were obliged to apply raising the possibility they were unlikely to be fully prepared or informed for the decision.

Both undergraduate and junior doctor experiences of radiology were significantly related to the timing of a career decision in our survey. Specifically, an undergraduate radiology project, obtaining career advice specific to radiology and regular attendance at departmental meetings were associated with an earlier decision (approximately 8 months sooner). Interestingly, *an undergraduate radiology project* and *obtaining career advice specific to radiology* are experiences that would have been instigated by the trainee and who therefore presumably already had some interest in the specialty. Notably, the only other early experience of radiology identified in the survey that would not have been instigated by the trainee – undergraduate tuition – was not frequently cited by respondents with nearly half having never received such tuition.

There was generally poor availability and/or uptake of early radiological experiences in our cohort. Postgraduate experiences were more frequently cited than undergraduate as influential in the process of making a career decision, a finding that has previously been reported across all medical specialties in the UK [5]. A worryingly large proportion of current trainees did not receive any undergraduate experience of radiology (neither tuition nor a radiology specific project). In addition, very few trainees received formal career advice, attended a radiology career open day or had the opportunity of experiencing radiology first hand as part of their Foundation Program. This is disappointing given that early radiology experience has been shown here and elsewhere to influence decision-making. A recent study compared U.S. students from two different years in the same medical school following implementation of radiology into the curriculum. Students exposed earlier showed increased interest and understanding of the specialty and a higher number subsequently considered radiology as a career [6]. Most of the radiology experiences cited as influential in career decision making by the respondents to our survey were obtained later in their training. For example, *day-to-day work and/or regular attendance at radiology meetings* as a junior doctor was the most influential factor in our cohort. There is therefore, a pressing need for UK undergraduate and early postgraduate medical training to include more radiology. A large body of work analyzing the factors which influence medical career choice across all specialties consistently demonstrates the need for early awareness of career options in undergraduate students and for maintenance of that interest with career guidance throughout the undergraduate period and beyond [7-11]. For example, policymakers for GP training in Australia have identified undergraduate electives as a key factor in enabling medical students to make an early and informed career choice in GP [7]. Paediatrics suffers a similar fate to radiology in that undergraduate experience of the specialty is usually only obtained in the senior years of medical school by which time many students may have already decided on an alternative career; it is recognized that encouragement in the uptake of *taster weeks* is hugely beneficial in these less mainstream specialties, particularly as relevant foundation posts are also likely to be very limited in number [8]. With the lowest number of applicants per post identified during the initial years of MMC [9], psychiatry struggles more than most specialties to recruit potential trainees. A large survey of UK medical students determined that those who had undertaken clinical placements in psychiatry were however more inclined towards the specialty [10].

### Limitations of the study

As with any research involving a voluntary survey, the greatest limitation in our work is the representation of our target cohort. At a little over 50% represented, there is a risk of misinterpretation of the data; ideally we would have liked nearer 70% or higher. We were unable to contact the potential participating trainees directly and had to invite them indirectly to our survey via the heads of training for each training scheme. This approach makes it impossible to ascertain which trainees did not complete the survey; ideally, non-responders would be targeted directly to improve the response rate. In addition, a small percentage of submitted questionnaires were incomplete. When creating the survey, the authors decided against making any questions mandatory as this can discourage participants from continuing with their submission. Our cohort is however, well spread throughout the UK training schemes with similar levels of representation for each and with none omitted. Demographically, the cohort is a good mix of gender and year of training, with no more than 15 trainees from any one medical school and with a significant inclusion from overseas medical schools. Although we must be cautious interpreting our data, we feel that it is nevertheless sufficiently robust to make the more generalized comments herein.

Another limitation of this work relates to the inclusion of only radiology trainees in our cohort. A broader picture of the effect of MMC on radiology applications would be obtained by surveying all Foundation Trainees, or at least, all applicants to radiology. Our approach enabled comparisons to be made between Traditional and Foundation Trainees but can make no comment on those applicants who were not successful at interview and may therefore have been more adversely affected by the changes imposed through MMC. Further work in this area might investigate differences between successful and unsuccessful applicants to radiology and would provide complimentary information to that which is presented here.

### The future of radiology training in the UK

In order for potential radiology trainees to make an early, truly informed choice and for the specialty as a whole to attract the best candidates, radiologists have to become more proactive with medical student training by offering appealing undergraduate projects and taking a greater role in undergraduate teaching, being prepared to give career advice, and providing the opportunity for students and junior doctors alike to see the workings of our radiology departments in both an informal and formal context. The RCR have identified career promotion as one of their primary objectives in a recently published strategic plan [12]. Major outlets for career promotion in the UK are the various training deaneries; however only one of these deaneries offers a radiology specific open day for potential applicants [13]. A few offer more generic career open days or actively encourage interested parties to make contact [14-17], while the remainder simply provide a relevant email address. In addition, undergraduate medical curricula should contain designated sessions in radiology. The RCR have also recognized the current problem of limited experience of the specialty for medical students and aim to encourage more radiology onto UK undergraduate curricula [18-20]. Encouragingly, our data show that Foundation Trainees were more likely to receive undergraduate experience of the specialty when compared with Traditional Trainees, i.e. things may be improving – the most recently graduated trainees in our cohort did obtain earlier experience of the specialty. However, Foundation Trainees were as likely as their *traditionally* trained counterparts to apply to specialities other than radiology through MMC. These data may represent ‘safety netting’ where multiple applications increase the chance of success, but it also implies that Foundation Trainees remain unsure of a career path at the time of their much earlier application; unfortunately, our survey may have enhanced this effect as it was performed during the tumultuous early period of MMC when there was significant uncertainty among applicants. Conversely and reassuringly, while trainees in our survey who did not feel ready to embark on a career in radiology had less time between graduating and starting training, this finding was not significant and did not predominantly affect Foundation Trainees who were as likely to be ‘very satisfied’ with radiology as a career.

The changes in UK postgraduate medical training brought in through MMC are here to stay. All specialties have to tailor their recruitment processes appropriately. It is no longer acceptable for potential radiologists to first experience the specialty as a spectator from the back of a darkened lunchtime meeting.

## Conclusions

• Historically, radiologists in the UK decided on their career 2.6 years after graduating

• MMC obliges trainees to apply for radiology approximately 1.5 years after graduating

• Early experiences of radiology allow trainees to make an earlier, informed career decision

• Respondents to our survey did not obtain sufficient early experience of the specialty; nearly half had no formal undergraduate radiology teaching, slightly fewer received no radiology specific career advice and postgraduate experiences were most commonly cited as influencing their career decision making

• Radiology should be included on all undergraduate medical curricula

## Methods

### Cohort sampled

In early 2009 there were 143 first and 201 second year radiology trainees in the UK who began training in August 2008 and August 2007, respectively. These 344 radiology STs were the first to enter training through the changes imposed by MMC. Due to preordained differences in the timing of their career decision, two distinct groups were defined within the cohort; *Foundation Trainees* are those who completed a Foundation Program and were therefore obliged to apply for speciality training during their second postgraduate year, while *Traditional Trainees* did not complete a Foundation Program and were not obliged to apply for specialty training at any particular time. The results from these two groups were compared.

The local independent research ethics service declared that as an evaluation of service development, this project did not require formal ethical review.

### Survey questionnaire

This cohort of trainees was invited via email to complete an anonymous online survey between November 2008 and February 2009. The survey was hosted on a web server; http://www.surveygizmo.com/ enables users to create their own surveys that can be accessed via a hyperlink in an email. Emails were sent indirectly to all trainees in our cohort; the Heads of Training for each of the 16 UK training deaneries (geographical division of UK training schemes) were contacted and asked to forward our email. To increase uptake, two waves of emails (separated by several months) were sent. To ensure the questionnaire was easily received and to identify any problems early, the survey was initially piloted on senior local trainees who were not to be included in the final sampled cohort.

To meet our stated study aims, the survey contained 36 questions designed to assess when and why trainees chose radiology (Results subsection – ‘Choosing Radiology’), whether MMC had any impact on the timing of their decision (‘Early Experience of the Specialty’ and ‘Factors Affecting Timing of Career Decision), and finally how satisfied they are with their choice and with the MMC process overall (‘Outcome of Decision to Train in Radiology’). Demographic data were also collected.

Answer styles for the questions included statements of fact, estimates based on personal experience and ranking on scales. Many questions also allowed the input of free text for further elaboration. To ease participant compliance and improve return rates, none of the survey questions were mandatory. The full questionnaire is provided as an appendix (see Additional file 1).

### Data analysis

The survey was closed one month after the final email invites were sent. Data were analyzed using SPSS (15.0) statistical software (Chicago, USA). Chi-squared testing was used for comparisons between dichotomous data. Simple t-tests were employed to compare normally distributed continuous and dichotomous data while Mann–Whitney U testing was employed where continuous data were skewed (age, years from graduation, satisfaction scores). To account for data clustering in relation to the medical schools attended by survey participants, Huber-White estimates were applied. Significance was taken as p < 0.05.

## Competing interests

The author(s) declare that they have no competing interests.

## Authors’ contributions

GM conceived concept, collected and analyzed data and drafted the manuscript. FE developed concept and critically revised manuscript. JTM developed concept and critically revised manuscript. All authors read and approved the final manuscript.

## Authors’ information

GM: Specialist Trainee on the South East Scotland radiology training program.

FE: Consultant radiologist based at the Western General Hospital in Edinburgh.

JTM: Consultant radiologist based at the Royal Infirmary of Edinburgh.

## Pre-publication history

The pre-publication history for this paper can be accessed here:

http://www.biomedcentral.com/1472-6920/12/93/prepub

## Supplementary Material

Additional file 1 Survey of UK Radiology Trainees in the Aftermath of ‘Modernising Medical Careers’. Click here for file

## References

[B1] Turner G Lambert TW Goldacre MJ Career choices for radiology: national surveys of graduates 1974-2002 from UK medical schools Clin Radiol 2006 61 1047 1054 10.1016/j.crad.2006.05.016 17097427

[B2] Donaldson SL Chief Medical Officer Unfinished business: proposals for reform of the senior house officer grade 2002 Crown Copyright http://webarchive.nationalarchives.gov.uk/+/http://www.dh.gov.uk/prod_consum_dh/groups/dh_digitalassets/@dh/@en/documents/digitalasset/dh_4071835.pdf

[B3] Education Board of the Faculty of Clinical Radiology Structured training in radiology 2004 Fourth The Royal College of Radiologists http://www.rcr.ac.uk/docs/radiology/pdf/STCR-2004.pdf

[B4] Education Board of the Faculty of Clinical Radiology Structured training curriculum for radiology 2007 The Royal College of Radiologists http://www.rcr.ac.uk/docs/radiology/pdf/Curriculum-CR-Jan2007.pdf

[B5] Watmough S Taylor D Ryland I Using questionnaires to determine whether medical graduates’ career choice is determined by undergraduate or postgraduate experiences Med Teach 2007 29 830 832 10.1080/01421590701551755 18236280

[B6] Brandstetter BF Faix LE Humphrey AL Preclinical medical student training in radiology: the effect of early exposure AJR 2007 188 W9 W14 10.2214/AJR.05.2139 17179333

[B7] Bunker J Shadbolt N Choosing general practice as a career: The influences of education and training Aust Fam Physician 2009 38 341 344 19458806

[B8] Goodyear HM Career guidance: how do we inspire students and young doctors to careers in paediatrics and child health? Arch Dis Child: Education and Practice Edition 2009 94 87 91 10.1136/adc.2008.141747 19460898

[B9] Fazel S Ebmeier KP Specialty choice in UK junior doctors: is psychiatry the least popular specialty for UK and international medical graduates? BMC Med Educ 2009 9 77 10.1186/1472-6920-9-77 20034389 PMC2805648

[B10] Budd S Kelly R Day R Student attitudes to psychiatry and their clinical placements Med Teach 2011 33 e586 e592 10.3109/0142159X.2011.610836 22022911

[B11] Straus SE Straus C Tzanetos K Career choice in academic medicine: systematic review J Gen Intern Med 2006 21 1222 1229 10.1111/j.1525-1497.2006.00599.x 17105520 PMC1924755

[B12] The Royal College of Radiologists Strategic plan 2011-2013 2011 The Royal College of Radiologists http://www.rcr.ac.uk/docs/about/pdf/rcr%2811%291_strategic-plan.pdf

[B13] Peninsula Radiology Academy Open Dayhttp://www.penra.org.uk/open-day

[B14] Mersey Deanery Medical Careers Fair 2012 http://www.merseydeanery.nhs.uk/careers2012

[B15] Northern Ireland Medical and Dental Training Agency NIMTDA Annual Careers Symposiumhttp://www.nimdta.gov.uk/2010/10/26/nimdta-annual-careers-symposium/

[B16] East of England Multi-Professional Deanery Radiology Training Programmeshttps://www.eoedeanery.nhs.uk/medical/page.php?page_id=447

[B17] East Midlands Healthcare Workforce Deanery Radiologyhttp://www.eastmidlandsdeanery.nhs.uk/page.php?id=829 10.1080/14739879.2015.1079023 26808795

[B18] Board of the Faculty of Clinical Radiology Shaping the future of interventional radiology 2007 The Royal College of Radiologists

[B19] The Royal College of Radiologists The place of clinical radiology and imaging in medical education: objectives, content and delivery of teaching 2008 The Royal College of Radiologists http://www.rcr.ac.uk/docs/radiology/pdf/medicalstudentpaper3.pdf

[B20] The Royal College of Radiologists Undergraduate Radiology Curriculum 2012 The Royal College of Radiologists http://www.rcr.ac.uk/docs/radiology/pdf/undergraduate_radiology_curriculum_2012.pdf

