# Supplementary material for: Survey of UK radiology trainees in the aftermath of ‘Modernising Medical Careers’
Source: BMC Med Educ. 2012 Oct 2;12:93. doi: 10.1186/1472-6920-12-93 (PMC3579711; doi:10.1186/1472-6920-12-93)
Supplement: Additional file 1 — Survey of UK Radiology Trainees in the Aftermath of ‘Modernising Medical Careers’. [file 1472-6920-12-93-S1.pdf]

National Survey of UK Radiology Trainees in the Aftermath of 'Modernising Medical Careers'

Additional File 1

The complete survey (HTML code removed):

MMC Survey

1. At what stage did you decide to train in radiology?

As a medical undergraduate

During my JHO or FY1 year

During my first SHO or FY2 year

During my second SHO year

During my third SHO year

Later... (please specify)

2. Did you have an alternative career plan prior to this?

No

Yes... (please specify)

3. During the MTAS/MMC process did you apply to any other specialties? (if appropriate please select more than one option)

None

Medical Specialty

Surgical Specialty

GP

Non-clinical

Other... (please specify)

4. If you did apply to other specialties, were you offered a training post?

N/A

No

Yes... (how many posts in total?)

5. Did you obtain a postgraduate qualification prior to entering radiology?

-- Please Select --

6. If you did obtain a postgraduate qualification, was this in part or complete?

Completed

Part 1

Part 2

Part 3

N/A

Undergraduate Experience of Radiology

7. Did you receive specific radiology training as an undergraduate?

(session taken by a radiologist where the primary focus was image interpretation)

No

Yes... (how many sessions in total?)

8. Did you undertake a student SSM (special study module)?

No

Yes

9. If you did, what subject area was this is in?

(if you can, please provide details for all SSMs undertaken)

10. As an undergraduate, did you complete an intercalated BSc?

No

Yes... (what subject?)

11. Did any of these experiences influence your eventual decision to train in radiology?

No

Yes... (which had the most impact?)

Contact with the Radiology Department as a Junior Doctor

12. As a junior doctor (FY1/FY2, JHO/SHO), how frequently did you attend departmental radiology meetings?

Never

Most days

Most weeks

One per month

Less frequently than once a month

13. Did you complete the foundation training programme?

No

Yes

14. If you trained as an FY2, did you undertake a diagnostics or radiology block?

No

Yes

N/A

15. Prior to submitting an application to a radiology training programme, did you formally spend any other time in your local radiology department?

(e.g. on a Taster Week)

No

Yes

16. Did any of these experiences influence your eventual decision to train in radiology? (answer no if you decided prior to this)

No

Yes... (which had the most impact?)

Other Experience of Radiology

17. At any time prior to entering radiology training were you involved in radiology research or audit?

No

Yes... (please elaborate)

18. Prior to entering your current training, did you attended a career open day for radiology, or obtain career advice specific to radiology?

No

Yes... (please elaborate)

19. Do you otherwise have experience within radiology by any means not described so far which might have influenced your career decision?

If yes, please elaborate

20. Did any of these experiences influence your eventual decision to train in radiology? (answer no if you decided prior to this)

No

Yes... (which had the most impact?)

Career Satisfaction - Remember, this survey is completely anonymous...

21. Through MTAS/MMC what position did you rank your current training deanery? *

First choice

Second choice

Third choice

Fourth choice

Other

22. How satisfied were you with the application process overall? *

Very disappointed Mildly disgruntled Indifferent Happy Delighted

23. Please feel free to elaborate on the above...

24. Did you feel adequately prepared when making your decision to enter radiology training? *

Yes

No... (why not?)

25. Are you happy with radiology as a career? *

No, I'm planning to change to something else   Unhappy, but unsure what to do about it   Not sure yet   Happy   Delighted

About You

26. What is your age? *

-- Please Select --

27. And your gender? *

Male

Female

28. From which medical school did you graduate? *

29. And in what year did you graduate? *

-- Please Select --

30. Which training deanery are you currently employed by?

-- Please Select --

31. And what year of training are you in currently? *

First Year

Second Year

Thank you once again for taking our survey
